# Mimicking CA3 Temporal Dynamics Controls Limbic Ictogenesis

**DOI:** 10.3390/biology11030371

**Published:** 2022-02-26

**Authors:** Davide Caron, Ángel Canal-Alonso, Gabriella Panuccio

**Affiliations:** 1 Enhanced Regenerative Medicine, Istituto Italiano di Tecnologia, 16163 Genova, Italy; davide.caron@iit.it; 2 BISITE Research Group, University of Salamanca, 37008 Salamanca, Spain; acanal@usal.es; 3 Institute for Biomedical Research of Salamanca, University of Salamanca, 37008 Salamanca, Spain

**Keywords:** temporal lobe epilepsy, deep brain stimulation, CA3, subiculum, interictal, brain slice, 4-aminopyridine, microelectrode array

## Abstract

**Simple Summary:**

Mesial temporal lobe epilepsy (MTLE) is the most common partial complex epilepsy in adults and the most often refractory to medications. Electrical deep brain stimulation (DBS) has proved effective in controlling seizures in animal models and in drug-refractory MTLE patients. However, there is still no unifying framework for DBS parameters, which are obtained by trial-and-error and based on arbitrary, fixed stimulation frequencies rather than on physiologically relevant patterns. Interictal activity may present the key to devising personalized and physiologically relevant DBS strategies. Interictal activity occurs between seizures and is a hallmark of the hyperexcitability of the epileptic brain; depending on the underlying mechanisms and site of origin, it may promote seizures (pro-ictogenic) or dampen them (anti-ictogenic). In this work, we address the possibility of controlling seizure activity by means of electrical stimulation fashioned as a surrogate interictal pattern known to be anti-ictogenic. We show that this approach can effectively control seizure activity while delivering fewer electrical pulses than fixed-frequency stimulation. Thus, mimicking the temporal dynamics of an anti-ictogenic interictal pattern may represent a straightforward, personalized and more efficient DBS strategy to ameliorate drug-refractory epilepsy. Our work heralds a paradigm shift toward physiologically meaningful rather than arbitrary DBS parameters.

**Abstract:**

Mesial temporal lobe epilepsy (MTLE) is the most common partial complex epilepsy in adults and the most unresponsive to medications. Electrical deep brain stimulation (DBS) of the hippocampus has proved effective in controlling seizures in epileptic rodents and in drug-refractory MTLE patients. However, current DBS paradigms implement arbitrary fixed-frequency or patterned stimuli, disregarding the temporal profile of brain electrical activity. The latter, herein included hippocampal spontaneous firing, has been shown to follow lognormal temporal dynamics. Here, we present a novel paradigm to devise DBS protocols based on stimulation patterns fashioned as a surrogate brain signal. We focus on the interictal activity originating in the hippocampal subfield CA3, which has been shown to be anti-ictogenic. Using 4-aminopyridine-treated hippocampus-cortex slices coupled to microelectrode array, we pursue three specific aims: (1) address whether lognormal temporal dynamics can describe the CA3-driven interictal pattern, (2) explore the possibility of restoring the non-seizing state by mimicking the temporal dynamics of this anti-ictogenic pattern with electrical stimulation, and (3) compare the performance of the CA3-surrogate against periodic stimulation. We show that the CA3-driven interictal activity follows lognormal temporal dynamics. Further, electrical stimulation fashioned as a surrogate interictal pattern exhibits similar efficacy but uses less pulses than periodic stimulation. Our results support the possibility of mimicking the temporal dynamics of relevant brain signals as a straightforward DBS strategy to ameliorate drug-refractory epilepsy. Further, they herald a paradigm shift in neuromodulation, wherein a compromised brain signal can be recreated by the appropriate stimuli distribution to bypass trial-and-error studies and attain physiologically meaningful DBS operating modes.

## 1. Introduction

Epilepsy is a life-threatening progressive brain disorder causing uncontrolled activity of the brain [1]. It carries among the highest burden of disease [2] and significant social stigma [3]. Mesial temporal lobe epilepsy (MTLE) is the most frequent epileptic syndrome in adults and the least responsive to medications [4]. Electrical deep brain stimulation (DBS) may represent a treatment option to ameliorate the clinical condition of drug-refractory MTLE patients. However, there is still no unifying framework for DBS parameters, which are still obtained by trial-and-error and based on arbitrary, fixed stimulation frequencies rather than on physiologically relevant patterns.

In search of the optimal DBS strategy to control seizures, several in vitro, in vivo, and in silico studies have explored a variety of stimulation paradigms, including open-loop (no feedback; in vitro—[5,6]; in vivo—[7,8,9,10,11,12]) and closed-loop (feedback-based) stimulation (in vitro—[13]; in vivo—[14,15]; in silico—[16,17]). Further, several clinical trials have addressed frequency and site of stimulation in open-loop [18,19] and closed-loop (responsive) DBS [20,21,22]. However, most of these studies were based on arbitrary stimulation policies that were not grounded in physiologically meaningful stimulation patterns. Instead, it would be desirable to attain natural entrainment of brain networks to re-establish dynamics that resemble the physiological working mode of the brain. In this scenario, interictal activity may present the key to devising personalized and physiologically relevant DBS strategies. Interictal activity occurs between seizures and is a hallmark of the hyperexcitability of the epileptic brain; depending on the underlying mechanisms and site of origin, it may promote seizures (pro-ictogenic) or dampen them (anti-ictogenic) [23,24,25]. Thus, DBS fashioned as a surrogate anti-ictogenic interictal pattern may represent a new avenue in DBS for drug-refractory epilepsy. In fact, while optimizing DBS parameters is a long-standing challenge, re-creating a missing brain signal may represent a straightforward strategy that would enable to bypass the need of demanding trial-and-error studies. To this end, understanding the temporal distribution of anti-ictogenic interictal activity is the very first step. In this regard, compelling evidence has demonstrated the existence of lognormal motifs in the brain structure and function. Specifically, it has been shown that the temporal profile of brain patterns exhibits skewed and heavily-tailed distributions with asymmetric variations around their mean, which can be described by a lognormal function [26]. Relevant to MTLE is the observation that hippocampal principal neurons exhibit lognormally-distributed firing in healthy conditions [27]. The hippocampus is indeed a key player in MTLE and it is often the target of stimulus delivery in DBS studies for MTLE treatment in the clinical setting [28,29,30,31,32,33,34,35,36], as well as in the preclinical setting in vivo [7,8,9,10,11,12,37] and in vitro [5,6,13,17]. Most importantly, in vitro studies have demonstrated that the interictal activity originating in the hippocampal subfield CA3 and recurring at ~1 Hz exerts an anti-ictogenic function when the hippocampal loop is intact; however, this function is hindered by hippocampal damage, which is also typically observed in drug-refractory MTLE patients [5,38,39,40]. Thus, exploiting the temporal profile of the CA3-driven interictal pattern may represent a meaningful and effective DBS strategy to ameliorate drug-refractory MTLE. In support of this view, in vitro studies have shown that electrical stimulation at 1 Hz, i.e., the average frequency of the CA3-driven interictal activity, can effectively control ictal activity when delivered in the CA1/subiculum, i.e., downstream to the site of hippocampal loop disruption [5,6]. Notably, low-frequency periodic stimulation delivered in the hippocampus has also been shown to effectively control seizures in epileptic rodents in vivo [9,12,37]. Nonetheless, if the CA3 preserved its lognormal temporal dynamics despite MTLE, periodic stimulation would fail to mimic the temporal profile of such a fundamental player in seizure control; in turn, it would not exert a natural entrainment of limbic networks, while possibly overstimulating brain tissue. However, whether the CA3-driven interictal activity also follows lognormal temporal dynamics remains unaddressed.

Here, we present a network electrophysiology study using rodent hippocampus-cortex (CTX) slices treated with the convulsant drug 4-aminopyridine (4AP) and coupled to microelectrode array (MEA), wherein we sought to: (1) address whether lognormal temporal dynamics can still describe the CA3-driven electrical pattern in the pathological condition (ictogenesis) as reported in the physiological condition, (2) explore the possibility of mimicking, with electrical stimulation, the temporal dynamics of the anti-ictogenic pattern generated by the CA3 to restore the non-seizing state in the disrupted hippocampal loop, and (3) compare the CA3-surrogate versus periodic stimulation in terms of efficacy (seizure reduction) and efficiency (number of delivered pulses).

We show that the CA3-driven interictal activity can be well described by lognormal temporal dynamics. Further, electrical stimulation fashioned as a surrogate CA3 interictal pattern can effectively control limbic ictal activity with statistically similar efficacy to fixed-frequency stimulation. However, the surrogate stimulation pattern uses fewer electrical pulses (higher efficiency). Projection analysis using the distribution parameters of the CA3-driven interictal pattern indicates that the patterned (CA3-surrogate) stimulus train may achieve higher efficiency than periodic pacing after a variable stimulus duration, reflecting the inter-individual variability of the distribution parameters.

The novelty of the proposed paradigm stems from its straightforward strategy: contrary to current DBS approaches, it does not entail trial-and-error fine-tuning since it directly exploits the temporal dynamics of brain patterns of the individual. In this, the relevance of the proposed paradigm is two-fold; first, adequately distributed pulse trains allow preserving the temporal profile of brain dynamics, thus providing a more natural way of modulating brain activity; second, and equally important, the foreseen reduction in the number of delivered pulses is expected to prolong the battery life of the DBS apparatus while decreasing the brain tissue stress. Thus, its implementation is expected to improve both open-loop and closed-loop DBS policies, including responsive stimulation and more complex designs based on machine learning techniques.

## 2. Materials and Methods

### 2.1. Brain Slice Preparation and Maintenance 

Brain slices 400 μm thick were prepared from male CD1 mice 4–8 weeks old. Animals were decapitated under deep isoflurane anesthesia, their brain was quickly removed and placed into ice-cold (~2 °C) sucrose-based artificial cerebrospinal fluid (sucrose-ACSF) composed of (mM): Sucrose 208, KCl 2, KH_2_PO_4_ 1.25, MgCl_2_ 5, MgSO_4_, CaCl_2_ 0.5, D-glucose 10, NaHCO_3_ 26, L-Ascorbic Acid 1, Pyruvic Acid 3. The brain was left to chill for ~2 min before slicing in ice-cold sucrose-ACSF using a vibratome (Leica VT1000S, Leica, Germany). Brain slices were immediately transferred to a submerged holding chamber containing room-temperature holding ACSF composed of (mM): NaCl 115, KCl 2, KH_2_PO_4_, 1.25, MgSO_4_ 1.3, CaCl_2_ 2, D-glucose 25, NaHCO_3_ 26, L-Ascorbic Acid 1. After at least 60 min recovery, individual slices were transferred to a submerged incubating chamber containing warm (~32 °C) holding ACSF for 20–30 min and subsequently incubated in warm ACSF containing the K^+^ channel blocker 4-aminopyridine (4AP, 250 μM), in which the MgSO_4_ concentration was lowered to 1 mM (4AP-ACSF [41]). Brain slice treatment with 4AP is known to enhance both excitatory and inhibitory neurotransmission and induce the acute generation of epileptiform discharges [42]. All brain slices were incubated in 4AP-ACSF for at least 1 h before beginning any recording session. All solutions were constantly equilibrated at pH = ~7.35 with 95% O_2_/5% CO_2_ gas mixture (carbogen) and had an osmolality of 300–305 mOsm/Kg. Chemicals were acquired from Sigma-Aldrich, Milano, Italy. 

All procedures have been approved by the Institutional Animal Welfare Body and by the Italian Ministry of Health (authorizations 860/2015-PR, approval date 24 August 2015, and 176AA.NTN9, approval date 20 October 2018), in accordance with the National Legislation (D.Lgs. 26/2014) and the European Directive 2010/63/EU. All efforts were made to minimize the number of animals used and their suffering.

### 2.2. Microelectrode Array Recording 

Extracellular field potentials were acquired through a 6 × 10 planar MEA (Ti-iR electrodes, diameter 30 μm, inter-electrode distance 500 μm, impedance < 100 kΩ). To allow for laminar flow and a high exchange rate of the 4AP-ACSF, a custom-made low-volume (~500 μL) recording chamber replaced the default MEA ring (cf. [41]). Individual slices were quickly (<60 s) transferred onto the recording chamber, where they were held down by a custom-made stainless steel/nylon mesh anchor. Slices were continuously perfused at ~1 mL/min with 4AP-ACSF, equilibrated with carbogen. Recordings were performed at 32 °C, achieved with the use of a heating canula (PH01) inserted at the recording chamber inlet port (temperature set at 37 °C) along with mild warming of the MEA amplifier base (temperature set at 32 °C), both connected to a TC02 thermostat. The recording bath temperature was checked using a k-type thermocouple. In the first series of experiments, signals were acquired with a MEA1060 amplifier using the Mc_Rack software; signals were sampled at 2 kHz and low-passed at half the sampling frequency before digitization. In the second series of experiments, signals were acquired with a MEA2100-mini-HS60 amplifier using the Multichannel Experimenter software; the amplifier was connected to the IFB v3.0 multiboot interface board through the SCU signal collector unit; signals were sampled at 2–5 kHz and low-pass filtered at 1–2 kHz before digitization. In all experiments, signals were stored in the computer hard drive for offline analysis. The custom recording chamber was designed and made by Crisel Instruments, Roma, Italy. The equipment for MEA recording and temperature control were purchased from Multichannel Systems (MCS), Reutlingen, Germany.

### 2.3. Electrical Stimulation

For electrical stimulation experiments, only partially disconnected brain slices were used (cf. [40]). In these brain slices, the CA3 output to the CTX was prevented by Schaffer collaterals disruption (either during the slicing procedure or via knife cut), whereas the CA1/subicular projections to the CTX as well as the CTX projections to the dentate gyrus were preserved. Stimuli were delivered in the CA1/subiculum and consisted of square biphasic direct current pulses conveyed via MEA electrode pairs connected in a bipolar configuration (±200–350 μA, balanced charge, 100 μs/phase, positive phase first). In the first series of experiments, pulses were delivered through the STG2004 stimulus generator controlled by the MC_Stimulus II software (both from MCS), whereas in the second series of experiments, pulses were delivered through the built-in stimulus generator of the MEA2100 system. A fast input/output (I/O) curve was always performed before the first stimulation protocol to find the current amplitude that would reliably elicit an interictal discharge (≥80% response rate, cf. [41]). The best-performing electrode pair and stimulus amplitude were then kept constant for each brain slice throughout the experiment. 

Stimulus trains mimicking the CA3-driven interictal pattern were generated ad hoc for each brain slice as a surrogate distribution described by the same mean (μ) and standard deviation (σ) of the biological inter-event interval (IEI) distribution (cf. Statistical analysis). To this end, we used the lognormal random number generator (RNG) function available in MATLAB 2016b (The Mathworks, Natick, MA, USA), wherein the seed was fixed across experiments for reproducibility. 

To validate the CA3-surrogate stimulation, we compared its performance against two periodic pacing protocols: (i) periodic pacing at 1 Hz, which has been demonstrated to effectively decrease limbic ictal activity [5,6], and thus served as a positive control; (ii) the matched periodic pacing protocol, sharing the same μ parameter of the CA3-surrogate stimulation, while having σ = 0 (i.e., no variations in the inter-pulse intervals). The stimulation protocols were generated in ASCII file format and imported into the MC_Stimulus II or the Multichannel Experimenter software. Performance indicators were defined as follows: (i) efficacy—the degree of ictal activity reduction; (ii) efficiency—the number of pulses used to achieve such a reduction. 

**Figure A1:**
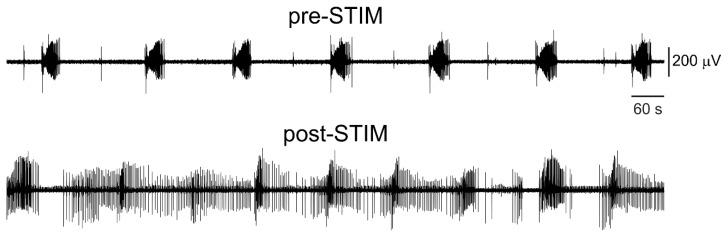
Partial recovery following electrical stimulation. Representative recording from the CTX of one brain slice in which ictal activity did not recover upon stimulus (STIM) withdrawal. In the pre-STIM condition, the brain slice generates recurrent, full-blown ictal discharges, along with a few slow interictal events. In the post-STIM period, the CTX no longer generates such events; the electrical pattern is instead made of barrages of slow interictal events and clusters of population bursts representing clear deteriorated ictal activity.

Baseline activity in the absence of stimulation was always recorded before and after each stimulation protocol and is overall referred to as control (CTRL). Post-stimulus baseline recording served both as the recovery phase and as the reference baseline activity for the subsequent stimulation protocol. Visually evident deterioration of ictal activity after electrical stimulation could indicate poor brain slice viability or an undesired OFF-stimulation suppression effect. Although the latter has been shown to be reversible within a few minutes of stimulus withdrawal, both in vitro [43,44] and in vivo [45], the sole evaluation of electrophysiological activity does not permit distinguishing it from brain slice deterioration. Thus, in the case of visually evident changes in ictal activity after stimulation, the experiment was not pursued. This happened in ~18% of the cases (cf., Figure A1 for a representative example). In the retained experiments, recovery of ictal activity was statistically assessed a posteriori, during offline analysis. 

The duration of each stimulation session was established based on a reference value of at least 3 times the mean interval of occurrence of ictal discharges (as observed from the first CTRL phase). A minimum stimulus duration of 20 min was set to standardize the experimental dataset. The established duration was kept constant throughout the experiment. All brain slices received a 20-min stimulus train, except for 1 brain slice, which required 30-min stimulation to meet our criteria, due to a slower occurrence of ictal events. Further, when noted, stimulation protocols were shuffled to exclude cumulative effects.

### 2.4. Surrogate Data Generation

To validate the contribution of increasing σ values on the sparseness of lognormal inter-pulse intervals and thus on the total number of pulses, 10 trials of pulse trains were generated for each σ value, changing the seed at each trial of the same σ, while keeping it constant across the different σ values.

### 2.5. Data Analysis

Electrode mapping relative to the areas included in the brain slice was performed using a custom GUI written in MATLAB R2016b [41]. Analysis of field potentials was performed offline using custom software written in MATLAB R2016b. To this end, signals were first low-pass filtered at 200 Hz using a 3rd order Butterworth filter to isolate field potentials by excluding possible contaminant multi-unit activity (MUA), which contribute high-frequency components > 200 Hz. Although recording with planar MEA from brain slices does not typically yield MUA, low-pass filtering was necessary to ensure isolation of field potentials for the appropriate detection of the CA3-driven interictal events since the detection of multiple MUA peaks within the same event would have biased the distribution statistics.

IEI distributions of CA3-driven interictal events were computed for each brain slice as follows: (i) in disconnected brain slices, which generate ictal events, we have analyzed at least three isolated interictal periods free of re-entrant ictal activity; (ii) in connected brain slices, we have analyzed a single interictal segment, since they did not generate ictal activity. In both cases, the overall segment duration was set to at least 10 min. In disconnected brain slices, we chose to exclude periods of re-entrant ictal activity for practical and conceptual reasons: on the practical level, the amplitude of re-entrant ictal activity could mask the CA3-driven interictal events, making it difficult to dissect it from ictal discharges; on the conceptual level, interictal periods free of re-entrant ictal activity better resemble the scenario of the intact hippocampal loop wherein the CTX cannot generate ictal activity. For simplicity, events timing was retrieved using a threshold-crossing peak-detection algorithm, where the threshold value was set at approximately half the amplitude of the signal. Binning used the Freedman–Diaconis algorithm to account for heavily-tailed distributions. The lognormal fit of the IEI distributions was performed using the maximum likelihood estimation method and the goodness of fit was evaluated by the adjusted R^2^ and the sum of squared error (SSE).

Ictal events were defined based on tonic-like or tonic-clonic-like signal features, disregarding the strict duration cut-off criteria commonly used by other works in the field. In fact, during ongoing electrical stimulation, emerging short population bursts could differ from interictal events observed during baseline recording and also from the evoked responses seen during the fast I/O. The borderline electrographic features of these events could represent hyperexcitable responses, prolonged interictal discharges, or entrained ictal events. Further, at the onset of the stimulation protocol, an ictal discharge could be triggered, as it may be expected, while stimulating hyperexcitable tissue before the network settles to a stably entrained state. These events were included in the analysis, regardless of their duration, and regardless of their spontaneous or electrically-evoked origin. This conservative approach was adopted to minimize the false negative bias that could lead to an overestimation of the stimulation efficacy.

**Figure A2:**
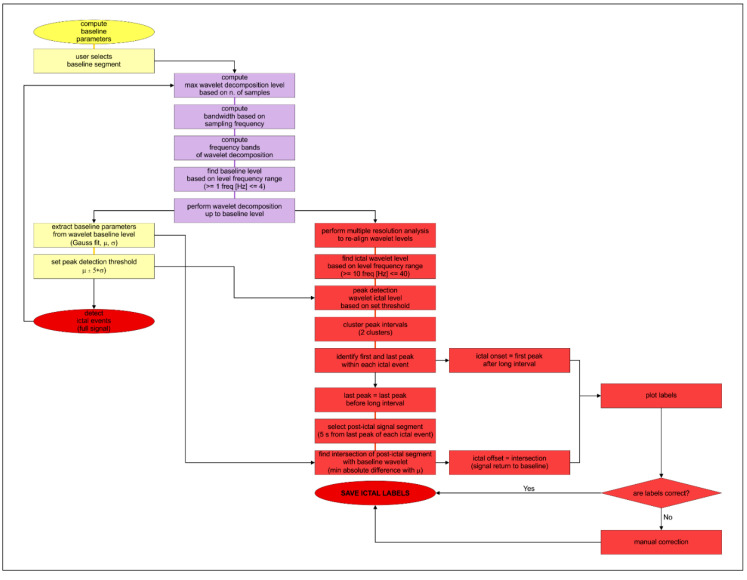
Seizure detection algorithm workflow. The color-coded blocks indicate the routines used for the extraction of the baseline parameters (**yellow**), the wavelet-based seizure detection (**red**), and the wavelet decomposition shared by the two (**violet**).

For the CTRL phases, ictal activity was identified using an automated seizure detection algorithm written in MATLAB 2016b, based on Daubechies discrete wavelet transform, type 4 [46]; detected events were always visually inspected and confirmed or manually corrected, as applicable. The wavelet level corresponding to a frequency range of 1–4 Hz was selected to compute the baseline parameters, whereas the level corresponding to 10–40 Hz was selected for ictal events detection. The algorithm flowchart is depicted in Figure A2. Briefly, the algorithm proceeded as follows: (i) extraction of the baseline parameters from a user-selected signal segment free of events: Gaussian fit of the baseline wavelet level; (ii) wavelet decomposition of the full signal, followed by multiple resolution analysis to re-align the obtained signal components; (iii) peak detection of the ictal wavelet level, based on a hard threshold set at μ ± 5 × σ (from baseline parameters); (iv) peaks clustering to identify ictal onset and termination (first and last deflection from baseline, respectively); (v) plot the event labels for visual inspection; (vi) manual correction, as applicable. 

For the electrical stimulation phases, the wavelet-based algorithm did not prove robust enough due to the presence of stimulation artifacts. Thus, events were manually labeled to ensure that any type of pathological discharge was identified as such. 

**Figure A3:**
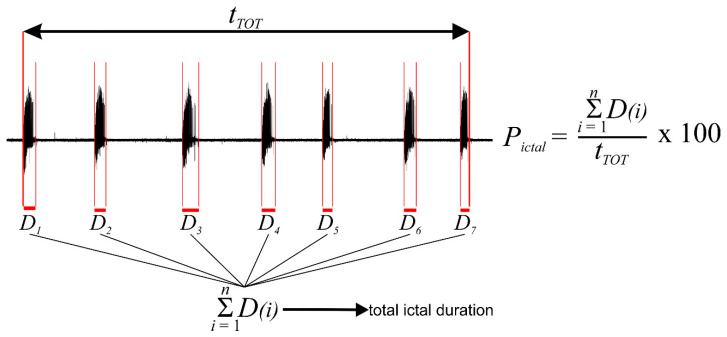
Method for the computation of *t_TOT_* and *P_ictal_*. Representative CTRL recording including seven ictal discharges (*D_1–7_*). The onset and termination of each ictal event are marked by the vertical red lines. The start and end of the observation window (*t_TOT_*) are marked by the higher thick red lines and indicated by the horizontal double-arrowed line. The duration of each measured ictal event (horizontal red line below the corresponding *D_1–7_* event) is summed to obtain the total time spent in the ictal state. This is then normalized against the *t_TOT_* and multiplied by 100 to obtain the percentage value (*P_ictal_*).

To quantify the labeled events, we computed the time-percentage spent in the ictal state (*P_ictal_*) during each recording phase (cf. [13,47]). Here, the term ictal state encompasses the broad definition of pathological events described above. Briefly, the pooled duration of all labeled events was normalized to the overall duration of the observation window (*t_TOT_*) and transformed to a percentage value, to yield:

$$P_{ictal}=\frac{\sum_{i=1}^{n}D\left(i\right)}{t_{TOT}}\times 100$$

where *i* is *i*th ictal discharge, *n* is the last measured ictal discharge, *D* is the ictal discharge duration and *t_TOT_* is the total observation time. For the stimulation phases (STIM), *t_TOT_* is the duration of the stimulation protocol; for the CTRL phases (no stimulation), *t_TOT_* is the time between the onset of the first and the termination of the last labeled event (Figure A3). Values of *t_TOT_* during CTRL were chosen to match as closely as possible to that of the respective STIM phase. As opposed to measuring the average event duration and interval of occurrence as distinct parameters, this method avoids biasing the statistics on the effect of electrical stimulation, should a single pathological event occur. In fact, in such a case, the event interval would not be measurable, whereas the event duration could falsely appear to be unchanged.

Ictal event amplitude was computed as the maximum peak-to-peak absolute value of labeled events. To this end, we chose a subset of six electrodes located along the antero-posterior axis of the CTX. The selected electrodes were kept constant across measured experimental phases within each brain slice to avoid biasing the measurement since the amplitude of field potentials depends on the electrode location with respect to sinks and sources.

### 2.6. Statistical Analysis

Throughout the text, *n* indicates the number of the specified samples. Normally distributed data are expressed as arithmetic mean ± SEM. Lognormally distributed data are described by their geometric mean and geometric standard deviation. For simplicity, we refer to these moments as μ and σ, regardless of the distribution type. 

Statistical analysis was performed using either custom MATLAB software, Origin Pro 20, or SPSS 20 (IBM, Armonk, NY, USA). Data were first checked for normality (Shapiro–Wilks test) and homoskedasticity (Levene’s test). Comparison of the efficacy of different stimulation protocols was performed using one-way ANOVA followed by the Games–Howell post hoc test (statistical significance set to a *p* < 0.05). 

To compare the number of pulses delivered by two stimulus trains, we used the two-sample Kolmogorov–Smirnov test. Specifically, data were first standardized using the formula:

$$z_{i}=\frac{x_{i}-\mu }{\sigma }$$

where *z_i_* is the standardized *i*th value, *x_i_* is the original value, and μ and σ are the mean and standard deviation of the dataset, respectively. Next, we first addressed whether the transformed data (representing the overall number of pulses delivered by the compared stimulus trains) were statistically different. Finally, in the case of a statistically significant difference, we compared the two stimulus trains proceeding backwards until the first time point of significant divergence. The basic assumption of this procedure is that the number of pulses delivered by a stimulus train increases over time (cumulative trend); as the Kolmogorov–Smirnov test compares the maximum difference between cumulative curves, the time point of significant difference will correspond to the last considered time point (maximum number of pulses in both stimulus trains). 

Throughout the text and figures, the *p* values are indicated as follows: * *p* ≤ 0.05; ** *p* ≤ 0.005; *** *p* ≤ 0.0001.

## 3. Results

### 3.1. Epileptiform Activity Induced by 4-Aminopyridine in Hippocampus-Cortex Slices

**Figure 1:**
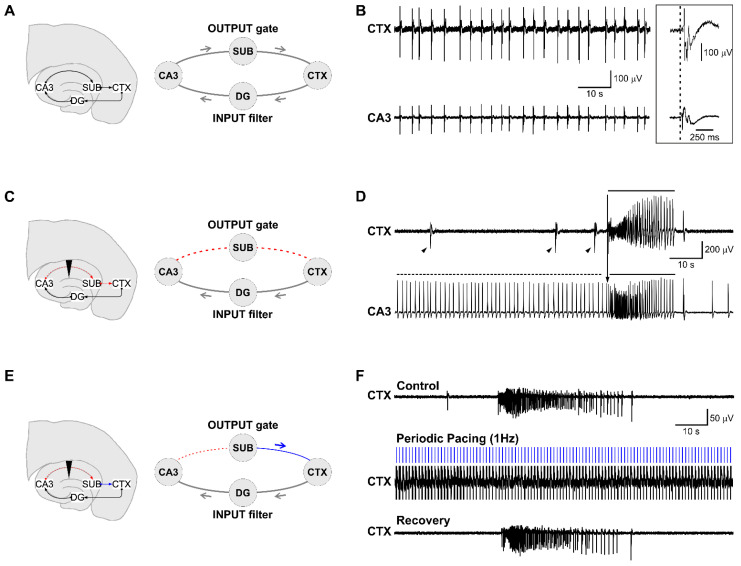
Network interactions in limbic ictogenesis in vitro. Epileptiform patterns induced in mouse brain slices by 4AP and recorded with MEA. (**A**) Intact brain slice. Schematic representation of the hippocampus-CTX slice and corresponding circuit diagram (**B**) Representative MEA recording showing the absence of ictal activity and the origin of the fast interictal pattern in the CA3 (boxed insert). (**C**) Partially disconnected brain slice schematic and corresponding circuit diagram. The Schaffer collaterals disruption is indicated by the dashed red line and the downward triangle. (**D**) Representative MEA recording showing that ictal activity (solid line) is generated by the CTX and re-enters the hippocampus (downward arrow). The interictal activity comprises a slow (arrowheads) and fast CA3-driven pattern (dashed line, also illustrated in (**A**)), the latter here restricted to the CA3. (**E**) Partially disconnected brain slice schematic representation and corresponding circuit diagram. (**F**) Representative MEA recording showing abolishment of ictal discharges during periodic pacing at 1 Hz and re-establishment of ictal activity upon stimulus withdrawal. CA3: Cornu Ammonis 3; DG: dentate gyrus; SUB: subiculum; CTX: parahippocampal cortex.

Rodent horizontal hippocampus-cortex (CTX) slices comprise the essential limbic structures involved in MTLE and thus represent a valuable anatomical substrate to study it. Treatment of this brain slice preparation with the convulsant drug 4-aminopyridine is an established and extensively characterized model of limbic ictogenesis [24]. Figure 1 shows microelectrode array (MEA) recording in three different scenarios offered by this model. These scenarios recapitulate previous experimental work that has substantiated the anti-ictogenic role of the CA3-driven interictal activity [5]. Figure 1A shows the schematic of this brain slice preparation and the corresponding circuit diagram when the hippocampal loop is intact. In this case, only the ‘fast’ CA3-driven interictal pattern occurs, while ictal activity is absent (Figure 1B). The boxed insert shows one representative event visualized at a faster time scale to emphasize its origin in the CA3 and its propagation to the CTX (dashed vertical line). Figure 1C shows the schematic and corresponding circuit diagram of a brain slice in which the hippocampal loop is disrupted by damage of the Schaffer collaterals. In this case, the fast interictal activity generated by the CA3 (horizontal dashed line) can no longer reach the CTX; the latter generates ‘slow’ interictal discharges (arrowheads) along with ictal activity (solid line), which re-enters the CA3 (downward arrow). Figure 1E shows the schematic and corresponding circuit diagram of a brain slice in which the disruption of the hippocampal loop is functionally recovered by fixed-frequency stimulation at 1 Hz, mimicking the average frequency of the CA3-driven interictal pattern. Stimulation is delivered in the CA1/subiculum downstream to the Schaffer collaterals damage to drive cortical networks. As shown in Figure 1F, this strategy abolishes ictal activity, which recurs again upon stimulus withdrawal. Thus, treatment of this brain slice preparation with the convulsant drug 4AP induces three types of electrical patterns, which may be present alone or in combination, depending on the preservation or disruption of the hippocampal loop. Our work will hereafter focus on the CA3-driven anti-ictogenic interictal pattern and the possibility of deploying its temporal dynamics in a personalized stimulation paradigm to control ictogenesis in the disrupted hippocampal loop.

### 3.2. The CA3-Driven Interictal Activity Exhibits Lognormal Temporal Dynamics

**Figure 2:**
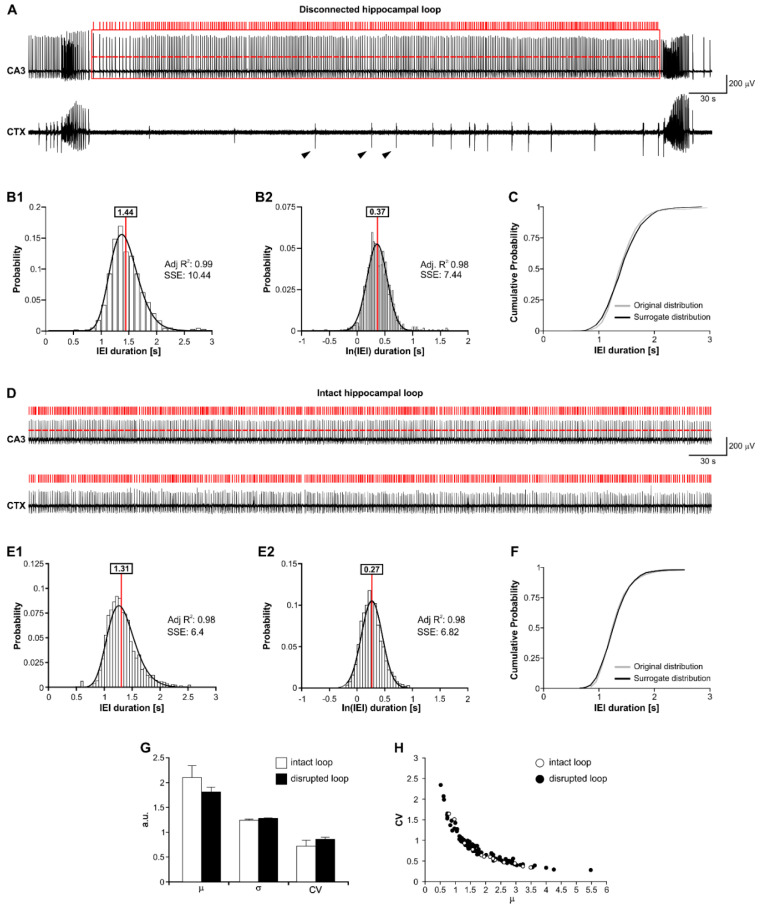
The fast CA3-driven interictal activity follows lognormal temporal dynamics. (**A**) Selection of one interictal period generated by the hippocampal subfield CA3 in a disconnected brain slice. (**B1**) Lognormal fit (solid line) of the IEI distribution from the experiment in (**A**). (**B2**) Gaussian fit (solid line) of the ln-transformed IEI (from (**B1**)). (**C**) The cumulative probability curve of ln-transformed IEI (from (**B1**)) is statistically similar to a surrogate normal distribution with the same μ and σ of the original data. (**D**) MEA recording of one interictal segment in a connected brain slice. (**E1**) Lognormal fit (solid line) of the IEI distribution from the experiment in (**D**). (**E2**) Gaussian fit (solid line) of the ln-transformed IEI (from (**E1**)). (**F**) The cumulative probability curve of ln-transformed IEI (from (**E1**)) is statistically similar to a surrogate normal distribution with the same μ and σ of the original data. (**G**) Distribution parameters of the IEI in connected and disconnected brain slices. (**H**) CV/μ relationship in connected and disconnected brain slices, exhibiting a similar power-law relationship.

First, we sought to determine whether the fast CA3-driven interictal pattern preserves the lognormal temporal profile reported in the healthy CA3 (cf. [27]). To this end, we analyzed the distributions of its inter-event intervals (IEI). Figure 2A shows a representative MEA recording of an interictal period between two consecutive ictal discharges obtained from the CA3 and the CTX in a brain slice in which the hippocampal loop is disrupted (hereafter termed disconnected brain slice). The red rectangle frames the interictal period and the red bars mark the detected fast interictal events, which are here restricted to the CA3. Note the absence of CA3-driven events in the CTX and the presence of slow interictal events (arrowheads).

Figure 2(B1) shows the inter-event interval (IEI) distribution obtained from this experiment (*n* = 1225 IEI from *n* = 3 interictal segments) and the corresponding lognormal fit (solid black line). As indicated in the insert, the IEI could be statistically well fit by a lognormal (μ = 1.44 s; σ = 1.25 s; adj. R^2^: 0.99; SSE: 10.44). In keeping with this, the corresponding normal distribution could well fit the ln-transformed IEI (μ = 0.37 s; σ = 0.22 s; adj. R^2^: 0.98; SSE: 7.44; Figure 2(B2)). If a population is lognormally distributed, its ln-transformed values are normally distributed. Based on this principle, the ln(IEI) distribution and a surrogate normal distribution of the same μ and σ parameters should be statistically similar. Thus, to further validate the lognormality of the IEIs, we compared their ln-transformed values against surrogate values randomly drawn from a normally distributed population of the same μ and σ (μ = 0.37 s; σ = 0.22 s). Statistical comparison of the empirical and theoretical datasets indicated their similarity (*p* > 0.05, Welch test). This is also emphasized by the nice overlap of the cumulative curves of the biological and theoretical IEIs, as shown in Figure 2C, after back-transformation to decimal values.

Figure 2D shows a representative recording of an interictal period from the CA3 and the CTX in a connected brain slice. The red bars mark the detected CA3-driven interictal events, which are here present in both regions. Figure 2(E1) shows the IEI distribution obtained from this experiment (*n* = 1402 IEI from *n* = 1 interictal segment) and the corresponding lognormal fit (solid black line). As indicated in the insert, the IEI could be statistically well fit by a lognormal (μ = 1.31 s; σ = 1.21 s; adj. R^2^: 0.98; SSE: 6.4). Again, the corresponding normal distribution could well fit the ln-transformed IEI (μ = 0.27 s; σ = 0.19 s; adj. R^2^: 0.98; SSE: 6.82; Figure 2(E2)). Figure 2F shows the validation of lognormality against a surrogate dataset randomly drawn from a normal distribution of the same μ and σ of the original dataset (μ = 0.27 s; σ = 0.19 s); again, statistical comparison of the empirical and theoretical datasets confirmed their similarity (*p* > 0.05, Welch test), as also emphasized by the nice overlap of the cumulative curves of the biological and theoretical IEIs, after back-transformation to decimal values. Lognormality was consistently observed across the analyzed disconnected and connected brain slices, i.e., regardless of the hippocampal loop integrity (connected brain slices—adj. R^2^: 0.96 ± 0.007; SSE: 22.05 ± 3.99; *n* = 648.38 ± 121.51 IEI; *n* = 8429 IEI overall; *n* = 13 brain slices from *n* = 12 mice. Disconnected brain slices—adj. R^2^: 0.96 ± 0.003; SSE: 24.79 ± 1.93; *n* = 761.48 ± 46.06; IEI; *n* = 70,867 IEI overall; *n* = 92 brain slices from *n* = 60 mice). Further, the distribution parameters were statistically similar between the two datasets (μ—connected brain slices: 2.11 ± 0.24 s, disconnected brain slices: = 1.84 ± 0.09 s; σ—connected brain slices: 1.25 ± 0.02 s, disconnected brain slices: 1.28 ± 0.01 s; *p* > 0.05 for each parameter; unpaired *t*-test; Figure 2G). In addition, the coefficient of variation (CV = σ/μ), which better represents the IEI variability around the μ parameter, was statistically similar in connected and disconnected brain slices (0.72 ± 0.12 and 0.85 ± 0.04, respectively; *p* > 0.05; unpaired *t*-test; Figure 2G). The similarity in the temporal profile of the CA3-driven interictal pattern in the intact and disrupted hippocampal loop is also emphasized by the overlapping CV/μ relationships shown in Figure 2H. Note that the CV/μ curve exhibits a power-law relationship (see Discussion). These results confirm that the temporal profile of the CA3-driven interictal pattern can be statistically well approximated by a lognormal.

### 3.3. Electrical Stimulation Fashioned as a Surrogate CA3-Driven Interictal Pattern Controls Limbic Ictogenesis

**Figure 3:**
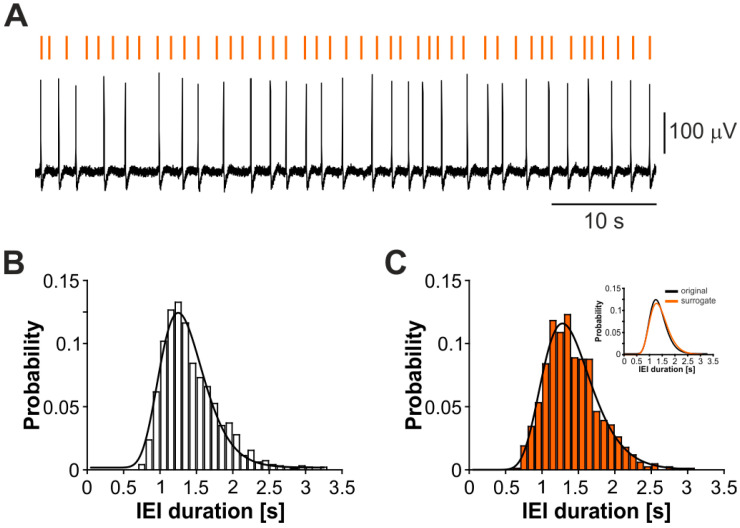
Surrogate lognormal pulse trains mimic the temporal dynamics of the CA3-driven interictal pattern. (**A**) Representative 60 s segment of the CA3-driven interictal activity (black) and the obtained surrogate pattern (orange bars). The probability distributions of the original (**B**) and surrogate (**C**) data, fit by a lognormal (solid lines), are statistically similar, as also emphasized by the overlapped fitted curves in the insert of panel (**C**).

Having established that the CA3-driven interictal pattern follows a lognormal temporal profile, we sought to determine whether mimicking its temporal dynamics could control CTX ictogenicity. In this series of experiments (*n* = 5 brain slices), periodic pacing at 1 Hz was always run at first as a positive control, since it is an established protocol to reliably restrain limbic ictal activity ([5,6,13]; cf. Figure 1). Further, periodic pacing at 1 Hz also served to assess the preservation of subicular projections to the CTX, as failure of this protocol is strongly indicative of the compromise of this output pathway by the slicing procedure. For lognormal stimulation, a surrogate distribution was generated for each brain slice, based on their interictal pattern in order to obtain an ad hoc stimulus train. Figure 3A shows a representative pulse sequence of 60 s (orange bars) compared to a representative segment of the original biological signal (black). The original IEI distribution from the same brain slice (Figure 3B) and the corresponding surrogate distribution (Figure 3C) are statistically similar (*p* > 0.05, two-sample Kolmogorov–Smirnov test), as also emphasized by the overlapped fitted distributions (Figure 3C, insert). Note that the term ‘similarity’ refers here to the statistical properties of the temporal profile of the biological and surrogate patterns, as described by the μ and σ parameters of their lognormal distribution; hence, while the biological and surrogate are described by statistically similar IEI distributions, it is expected that they are not identical in terms of events timings.

**Figure 4:**
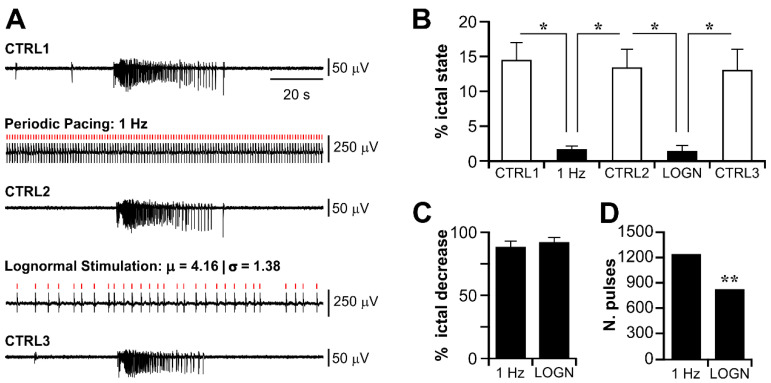
Surrogate lognormal pulse trains delivered in the subiculum control limbic ictal activity. Lognormal stimulation exerts an anti-ictogenic control similar to periodic pacing at 1 Hz. (**A**) Representative traces (120 s duration) for each experimental phase. Stimulus artifacts are partially removed by offline signal post-processing and the delivered pulses are marked by the red bars. In this experiment, both stimulation protocols abolished ictal activity. (**B**) Summary of the results statistics for *P_ictal_* during CTRL and stimulation. (**C**) The efficacy of ictal state reduction is statistically similar among the two stimulation protocols. (**D**) The theoretical number of pulses delivered by a 20-min lognormal stimulus train drawn from the population IEI distribution versus the number of pulses delivered by periodic pacing at 1 Hz of the same duration. The lognormal stimulus train consists of significantly less pulses than periodic pacing at 1 Hz. * *p* < 0.05; ** *p* < 0.005.

Figure 4A shows 120 s-segments of representative recordings from the CTX during each experimental session; stimulus timings are marked by red bars. In this experiment, both periodic pacing at 1 Hz and lognormal stimulation prevented ictal activity, which recurred again upon stimulus withdrawal. As summarized in Figure 4B, in this set of experiments, each stimulation protocol markedly decreased the overall ictal state duration (*P_ictal_*—CTRL1: 14.38 ± 2.67; STIM 1 Hz: 1.58 ± 0.5; CTRL2: 13.32 ± 2.79; STIM lognormal: 1.31 ± 0.85; CTRL3: 12.96 ± 3.1; one-way ANOVA, F(df): 10.86(4), *p* < 0.05 for each stimulation phase against the preceding control phase and the subsequent recovery phase). Moreover, the efficacy of the two stimulation protocols was statistically similar (% ictal state reduction—STIM 1 Hz: 87.57 ± 4.72; STIM lognormal: 91.39 ± 4.42; *p* > 0.05, paired *t*-test. Figure 4C). 

Table 1 reports the stimulation parameters and the number of pulses delivered by the lognormal and 1 Hz stimulus trains. 

From these data, it is also evident that the lognormal stimulation delivered strikingly less electrical pulses than periodic pacing at 1 Hz (*p* < 0.001 in all experiments; two-sample Kolmogorov–Smirnov test). This result is expected, since the μ and σ parameters of the lognormal pulse trains departed significantly from the descriptors of 1 Hz (lognormal: μ = 2.62 ± 0.62, σ = 1.4 ± 0.06; periodic 1 Hz: μ = 1; σ = 0). Thus, while lognormal stimulation exhibited the same efficacy (ictal state reduction) as periodic pacing at 1 Hz, it overall demonstrated a significantly higher efficiency (number of delivered pulses). Although in this subset of brain slices, the frequency of the CA3-driven interictal events was rather slower than what was reported in the literature (reference range value: 0.25–1.5 Hz—[5]; this work—range: 0.23–0.9 Hz, median: 0.34 Hz), the population distribution parameters (cf. Figure 2) also pointed to the possibility of adopting a more conservative stimulation approach. To validate this observation from the theoretical standpoint, we generated a surrogate lognormal pulse train of 20-min duration (i.e., the average stimulation duration during brain slice experiments) based on the pooled IEI statistics; then, we compared the number of pulses hypothetically delivered by the lognormal stimulus against those that would be delivered by periodic pacing at 1 Hz. As expected, the lognormal pulse train consisted of significantly less pulses than the periodic stimulus train at 1 Hz (Figure 4D; lognormal: 797; periodic 1 Hz: 1200; *p* < 0.005, two-sample Kolmogorov–Smirnov test). Thus, the latter might be too aggressive by delivering an excessive and unnecessary number of pulses. However, we could not exclude that, having systematically performed periodic pacing at 1 Hz before lognormal stimulation, we might have introduced an experimental bias consequent to an undesired OFF-stimulation suppression effect [44].

### 3.4. Lognormal Surrogate Pulse Trains Are More Efficient Than the Matched Periodic Stimulation

In light of the obtained results, we reasoned that a matched periodic pacing protocol, sharing the same μ parameter of the lognormal pulse train, would enable a direct comparison of the performance of the lognormal versus periodic stimulation. Thus, as the next logical step, we compared the efficacy and efficiency of lognormal stimulation against its matched periodic pacing protocol, and in this case, shuffling their sequence (*n* = 6 brain slices overall; *n* = 3 brain slices each shuffled stimulation sequence).

**Figure 5:**
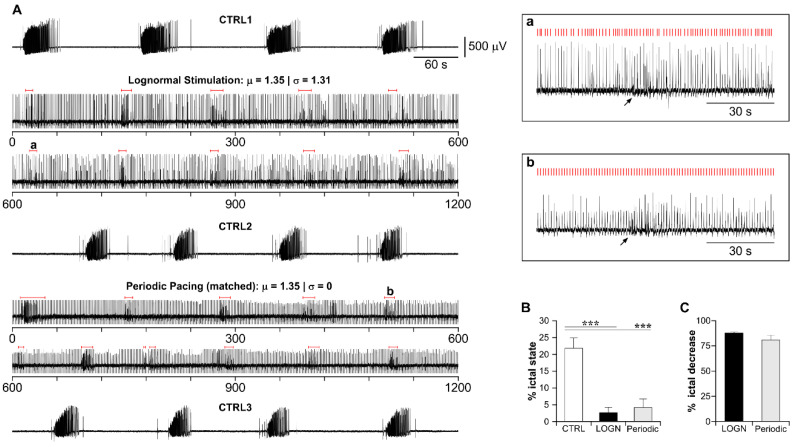
The surrogate lognormal pulse train is as effective as the matched periodic pacing protocol. (**A**) Representative experiment illustrating the effect of lognormal stimulation vs. its matched periodic pacing protocol (CTX recording). The signals for the stimulation protocols are the full 20-min session, split into two 10-min chunks for clarity. In this experiment, the CTX generated only short population bursts (red lines) during electrical stimulation. Ictal activity returned similar to the initial baseline condition (CTRL) after stimulus withdrawal. The boxed signals are the events marked by (**a**,**b**) in the stimulation sessions, visualized at a faster time scale. Stimulus times are indicated by the red bars. (**B**) Summary of the % of ictal state duration during CTRL (pooled data), lognormal and periodic stimulation. Both protocols significantly decrease the overall ictal state. (**C**) Comparison of the % of ictal state decrease of the lognormal and matched periodic pacing paradigms vs. pooled CTRL data. The two stimulation protocols exhibit a statistically similar efficacy. *** *p* < 0.0001.

Figure 5A shows the recordings from the CTX during each experimental session of a representative experiment. In this case, both lognormal stimulation and its matched periodic pacing protocol strongly decreased ictal activity, while only short population bursts could be observed (red lines). Representative instances of these pathological events visualized at a faster time scale are indicated by the arrows in the boxed inserts. These events likely represented a temporary increase in CTX excitability toward ictal transition, which was, however, aborted by the ongoing electrical stimulation. 

For the statistical comparison, we first addressed possible OFF-stimulation effects (or tissue deterioration) by comparing the ictal state duration and ictal event amplitude across CTRL phases. Population statistics indicated that both parameters were statistically similar among CTRLs (one-way ANOVA), *p* > 0.05 for each paired comparison; *P_ictal_*: F(df): 0.17(2); ictal event amplitude: F(df): 0.61(2); see Table 2). Thus, the efficacy of each stimulation protocol was compared against the pooled CTRL values since the stimulation protocols were shuffled.

As shown in Figure 5B, both lognormal and periodic stimulation markedly decreased the ictal state duration compared to the pooled CTRL (one-way ANOVA, F(df): 43.08(2); *p* < 0.0001 for each paired comparison; see Table 3). Further, as shown in Figure 5C, their efficacy (% ictal state reduction) was statistically similar (paired t-test, *p* > 0.05; see Table 3).

Table 4 reports the stimulation parameters and the number of pulses of the two stimulation protocols. Again, the lognormal stimulus train delivered overall less electrical pulses than periodic pacing; however, in this case, the difference in pulse count did not achieve statistical significance within the experimental test time (*p* > 0.05, two-sample Kolmogorov–Smirnov test).

**Figure 6:**
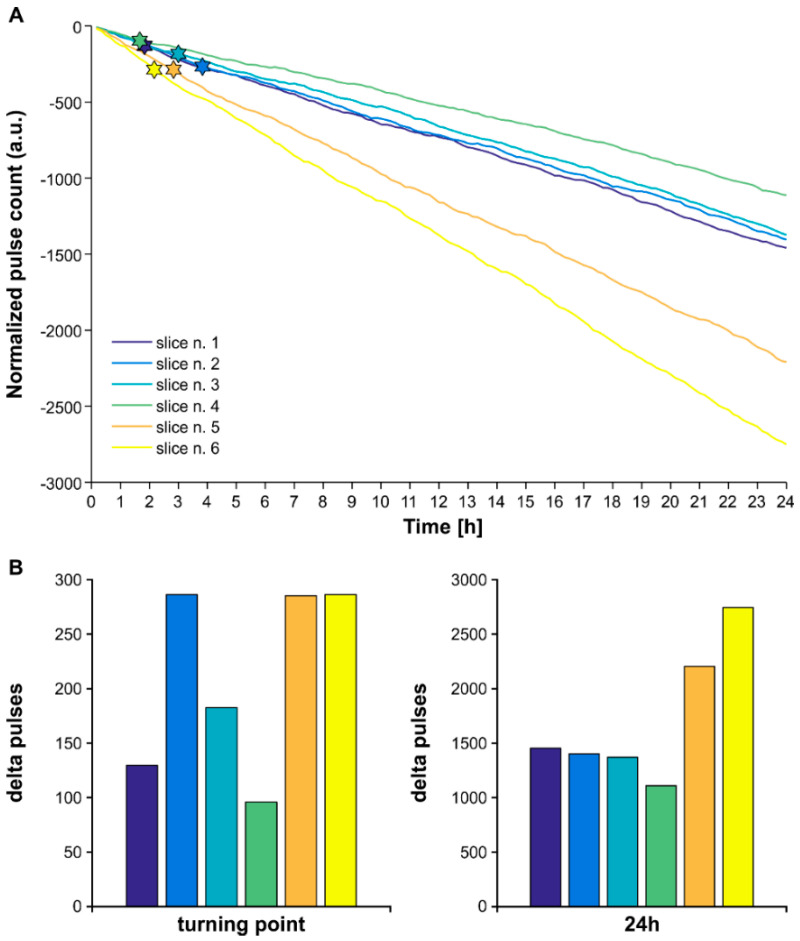
Higher efficiency of lognormal vs. periodic stimulation. (**A**) The cumulative trend of the normalized number of pulses delivered in 24 h by lognormal and matched periodic pulse trains; pulse distribution parameters obtained from the experimental dataset illustrated in Figure 5 (*n* = 6 brain slices). The start symbol indicates the time point of significant change in efficiency. (**B**) Count of excess pulses delivered by the periodic versus their respective lognormal stimulus trains at the efficiency turning point indicated in (**A**), and at 24 h. Color-coding as in (**A**).

Reasonably, the short duration of the two stimulus trains (20 min) did not suffice to unveil the higher efficiency of the lognormal versus periodic stimulation, since they shared the same μ parameter. Given that the number of delivered pulses increases over time (cumulative trend), we hypothesized that the lognormal stimulus train might require several hours to become statistically more efficient than its matched periodic pacing protocol. As brain slices do not enable such long-term electrophysiological recordings, we addressed this hypothesis on the theoretical level. To this end, we used the μ and σ parameters of the experimental dataset (*n* = 6 brain slices) to obtain six pairs of stimulus trains of extended (24 h) duration and compared the number of delivered pulses in 10-min steps. In keeping with the inter-individual variability of the μ and σ parameters, statistical comparison indicated that the stimulus trains departed significantly in the number of delivered pulses at variable time points (range time point: 100–250 min; range delta pulses: 96–287; *p* < 0.05; 2-sample Kolmogorov–Smirnov test). At 24 h, the periodic stimulus trains exceed their respective lognormal by >1000 pulses (range delta pulses: 1374–2752; *p* < 0.0001; 2-sample Kolmogorov–Smirnov test). These results are summarized in Figure 6. Panel A illustrates the performance of the lognormal stimulus trains as a cumulative trend; for clarity, the cumulative curves are normalized by the mean number of pulses delivered by the matched periodic pacing protocol; for each stimulus train, the star symbol indicates the time point of significant change in the efficiency. Panel B reports the raw values of excess pulses delivered by each periodic stimulus train at the time point indicated in panel A, and at 24 h. 

### 3.5. The Higher Efficiency of Lognormal Stimulation Reflects the Pulse Sparseness Determined by the Distribution Parameters

**Figure 7:**
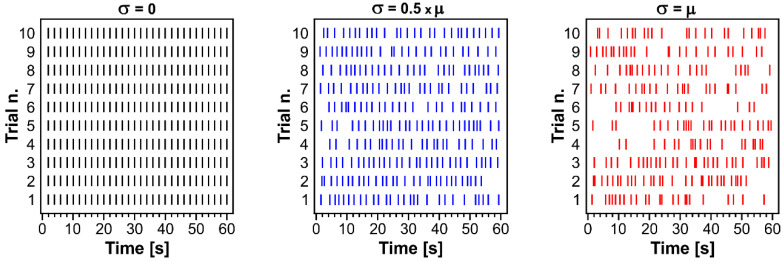
The higher efficiency of lognormal stimulation vs. periodic pacing resides in the degree of pulse sparseness. Computer-generated sequences of 60 s-pulse trains drawn from lognormal distributions with μ = ln(2) and increasing σ values (0, 0.5 × μ and μ), 10 trials each; at σ = 0, the resulting pulse trains are periodic (constant inter-pulse interval). At increasing σ values, the number of pulses becomes smaller due to the increased inter-pulse interval variability (σ), spanning an increasingly wide interval range.

We ascribed the higher efficiency of lognormal versus periodic stimulation to the variability of the inter-pulse intervals (σ) since the two stimulus trains shared the same μ parameter. Specifically, in the lognormal stimulus train σ > 0, whereas in the matched periodic stimulus train σ = 0. As the σ value increases, the inter-pulse interval variability becomes greater and so does the pulse sparseness. Thus, we reasoned that the σ value is inversely correlated with the number of pulses, i.e., the number of pulses decreases as σ increases. To validate this point, we generated 60 s-pulse trains in which μ was kept constant (μ = 2) while σ was varied among 0, 0.5 × μ and μ (Figure 7). 

These data confirmed that when σ = 0, pulses are generated in a periodic fashion (*n* = 30 pulses each trial, frequency: 0.5 Hz), while the sparseness of the pulses increases upon the increase in the inter-pulse interval variability (σ). In fact, when σ was set at 0.5 × μ, the same number of pulses generated by the periodic stimulus (*n* = 30) was generated in 2/10 trials only, for an overall mean frequency of 0.46 ± 0.01 Hz; when σ was set to be equal to μ, this was observed in 1/10 trials only, for an overall mean frequency of 0.39 ± 0.02 Hz. 

Overall, our work provides compelling evidence that electrical stimulation mimicking the temporal dynamics of an anti-ictogenic interictal pattern is a very effective and highly efficient open-loop stimulation approach to control seizures. This provides the grounds for a physiologically relevant and less aggressive DBS paradigm to treat drug-refractory MTLE and, possibly, other epileptic disorders.

## 4. Discussion

In this work, we have built upon two previous demonstrations pertinent to the activity of the hippocampal subfield CA3: (1) in animal models of acute limbic ictogenesis, the CA3-driven interictal activity exerts an anti-ictogenic function [5,6], and (2) in healthy animals, the CA3 activity follows lognormal temporal dynamics [27]. Based on these demonstrations, we have explored the possibility of preventing limbic ictal activity by means of open-loop electrical stimulation fashioned as a surrogate CA3-driven interictal pattern. To this end, we have characterized the temporal profile of the CA3-driven interictal activity and we have devised ad hoc open-loop stimulation strategies.

Our main findings can be summarized as follows: (1) the interictal activity generated by the CA3 preserves the lognormal temporal dynamics described in the same region in the healthy brain; (2) electrical stimulation of the CA1/subiculum fashioned as a surrogate CA3-driven interictal pattern controls seizure activity with similar efficacy but higher efficiency than periodic pacing.

### 4.1. The Temporal Dynamics of the CA3-Driven Interictal Activity Preserve the Lognormal Statistics of the Healthy Hippocampus

By merging experimental and theoretical approaches, we have provided compelling evidence that the electrical activity generated by the CA3 during acute ictogenesis preserves the lognormal temporal dynamics observed in healthy rodents [27]. Remarkably, we have also found that lognormality is preserved regardless of the hippocampal loop integrity; further, the temporal profile of the CA3-driven interictal pattern is statistically similar in the intact and disrupted hippocampal loop, as evidenced by the similarity of the IEI distribution parameters and the CV/μ relationships in the two conditions. Thus, the CA3-driven interictal activity may be interpreted as an exacerbation of naturally occurring dynamics, possibly in an attempt to halt ictogenesis. 

In the in vitro model used in this study, the CA3-driven interictal activity exerts an anti-ictogenic role when the intact hippocampal loop is challenged with convulsant agents. While this in vitro model addresses a specific epileptic syndrome, it points to the relevance of so-called ‘green’ interictal spikes observed in the EEG of epileptic patients farther away from the seizure onset zone. Green spikes seem to reflect a higher inhibitory brain activity, which possibly explains why seizures do not arise from nor tend to spread to brain areas where they are present [23]. These features support the view that green interictal spikes may be anti-ictogenic; thus, the CA3-driven interictal events may be regarded as ‘green’ [25]. From the translational perspective, it would be highly relevant to investigate the temporal dynamics of the interictal discharges generated by the CA3 and better define their role in epileptic rodents and in human MTLE patients. In a broader perspective, it is reasonable to hypothesize that the green interictal spikes may be the key for novel personalized treatments for epilepsy, including ad hoc open-loop DBS strategies (discussed below). In this regard, it will be crucial to identify specific electrographic biomarkers that could help dissect green from red interictal spikes beyond their anatomical location relative to the seizure onset zone.

### 4.2. Surrogate Brain Patterns Are a Feasible, Effective and Efficient DBS Strategy

Open-loop electrical stimulation based on surrogate CA3 interictal patterns proved highly effective in decreasing the overall ictal state duration. Noteworthy, we observed a striking difference in efficiency between lognormal and periodic stimulation, indicating that the latter approach might be too aggressive. The higher efficiency of patterned stimuli is likely due to the coexistence of two factors, which may overall contribute to an increased probability of neuronal network entrainment: (i) lognormal pulse train statistics better match the natural temporal dynamics of the CA3-driven interictal activity (cf. [27]) and its adaptation to ongoing ictogenic dynamics; (ii) the wide inter-pulse interval range of lognormally-distributed stimuli may allow neuronal network recovery between subsequent pulses; in turn, this may make neuronal networks more prone to respond to (and thus to be entrained by) electrical stimuli. In support of this view, it has been shown that entrainment of neural activity exhibits the highest reliability when stimuli possess power-law statistics, a phenomenon described both at the single-neuron [48] and at the neuronal network level [49]. As further emphasized by Buzsaki, G. and K. Mizuseki [26], power-law and lognormal distributions connect naturally, and brain dynamics may be equally well described by any of the two [50]. Remarkably, we have found that the CV/μ curve of the CA3-driven interictal pattern exhibits a power-law relationship (cf. Figure 2F). Thus, it is reasonable to hypothesize that the efficacy and efficiency of lognormally-distributed stimuli are contributed by the entrainment of limbic networks into the appropriate temporal regime. Further, since periodic stimulation neglects the natural statistics of brain patterns [26,51], it likely forces brain activity away from physiological temporal dynamics and thus it may not represent the ideal therapeutic approach. The relevance of devising DBS strategies that resemble brain patterns is also emphasized by increasing evidence of DBS effects beyond the stimulated brain area [52]. The view that temporally-distributed stimuli may outclass the performance of periodic stimulation is further witnessed by recent experimental DBS strategies, including Poisson pulse trains [7,10] and asynchronous multi-site stimulation [53] in epileptic rodents. The relevance of an appropriate pulse spacing for seizure control is also evidenced by the poor performance of periodic stimulation delivered at intervals of 5 s or longer, i.e., 0.2 Hz or lower [6]. In this regard, it is interesting to note that, in our work, the frequency of the CA3-driven interictal pattern exhibited the slowest values around 0.2 Hz (cf. Figure 2F) and so did the average frequency of the slowest lognormal stimulus train. In line with what was discussed above, we may hypothesize that, when the stimulus train is tailored around a physiologically relevant brain pattern, its efficacy may not suffer from specific frequency constraints. It also needs to be noted that the distributed stimuli used in the above-cited studies [7,10,53] were either arbitrarily chosen or arbitrarily drawn from a distribution, rather than reflecting the temporal dynamics of a relevant brain signal. This approach is fundamentally different from what was proposed here, and it further corroborates our view that DBS paradigms should be based on physiologically meaningful patterns. 

As opposed to single-site stimulation, an emerging concept in DBS is phase-shifted multi-site stimulation (‘coordinated reset’). This paradigm has demonstrated its greater performance against periodic pacing in controlling epileptiform activity and inducing long-lasting after-effects in hippocampal slices [54]. Thus, both the temporal profile and the spatial distribution of electrical stimulation appear to be highly relevant for DBS efficacy. Here, we have addressed the temporal distribution of stimuli delivered to a single site. As a step further, it would be highly relevant to bring the coordinated reset paradigm into our experimental model.

One limitation of our study was the short duration of the stimulus train and of the overall MEA recording, as imposed by the limited viability of brain slices in time. Thus, we could not address the effect of long-term (hours) electrical stimulation, nor the establishment of long-lasting after-effects. The latter have been related, among others, to the induction of plasticity phenomena, such as homeostatic plasticity and synaptic depression [55]. In this regard, it needs to be noted that low-frequency hippocampal DBS can rescue the otherwise impaired hippocampal plasticity in epileptic rodents [56,57]. Thus, it is reasonable to hypothesize that prolonged, low-frequency lognormal stimulation may favor DBS after-effects. In vivo studies in epileptic rodents will help elucidate this aspect.

### 4.3. The Subiculum Is a More Suitable DBS Target Than the Hippocampus Proper in MTLE Patients

The hippocampus represents one key target structure in DBS research for MTLE treatment, in light of its crucial involvement in epileptogenesis and ictogenesis. Here, we have delivered electrical stimulation in the subiculum, which is downstream to the hippocampus proper and the least anatomically damaged area in MTLE patients and animal models [58,59,60]. Further, the subiculum serves as the hippocampal output gate [61], and its epileptic hyperexcitability plays a pivotal role in the initiation and maintenance of limbic seizures [40,62,63,64,65]. In converging towards a more mechanistic understanding of DBS, its modulatory action is considered to unfold via entrainment of both local [66] and distal brain networks [52]. Thus, electrical stimulation delivered in the subiculum could represent a valid approach to bypass the sclerotic hippocampus, rectify the hyperexcitability of the hippocampal gate (local effect) and recruit efferent pathways to entrain distal CTX networks (global effect). In support of this view, several clinical studies in MTLE patients have reported variable therapeutic outcomes of hippocampal DBS, including non-responsiveness [28,29,30,32,33,34,35]. Hippocampal sclerosis has been brought about as a relevant factor hindering seizure reduction since sclerotic tissue seems poorly responsive to electrical stimulation; in these patients, a good therapeutic effect (>50% seizure reduction) was instead achieved by stimulating the subiculum, which is downstream of the sclerotic hippocampus proper [67]. Remarkably, direct stimulation of the seizure focus seems less effective than stimulation of afferent pathways via the subiculum [6,67]. Overall, these studies support the view that the subiculum may be a more suitable DBS target in MTLE patients. Clinical trials are currently ongoing to further investigate this possibility [68].

### 4.4. A Green Spikes-Based Grammar for DBS in Epilepsy

DBS is a promising approach to ameliorate epilepsy, but the best stimulation strategy has yet to be established: site and parameters of electrical stimulation strongly depend on the type of epilepsy and on the patient’s specific factors [69]. Experimental work performed in silico, in vitro, and in vivo as well as clinical trials has addressed the issue of inter-individual variability by proposing ad hoc stimulation policies, based on open-loop or closed-loop strategies. Whereas open-loop stimulation operates blind to the ongoing brain activity, closed-loop designs rely on brain feedback to deliver stimulation on-demand. While the scientific community is investigating the deployment of distributed stimuli, fixed-frequency stimulation is still the most used approach both in open-loop and closed-loop DBS, including the FDA-approved responsive neurostimulation (RNS) [22]. Thus, even the most advanced closed-loop design may be seen as a more flexible periodic pacing strategy. The present work proposes a straightforward strategy: exploiting individual-specific temporal features of a naturally anti-ictogenic pattern to re-establish the non-seizing state. This strategy heralds the possibility of bypassing the need for demanding trial-and-error approaches to optimize open-loop and closed-loop DBS parameters; further, it carries the advantage of being designed ad hoc on the individual’s brain dynamics rather than on population statistics. In this regard, our work provides the proof of principle of a new green spikes-based grammar as a starting point for personalized DBS.

## 5. Conclusions and Future Perspectives

In this work, we have built upon the experimental evidence of the anti-ictogenic role exerted by the fast interictal pattern generated by the hippocampal subfield CA3 to challenge two major drawbacks of the current DBS mindset: (i) the trial-and-error approach and (ii) the deployment of arbitrary stimulation parameters, affecting both periodic and distributed stimulation. Specifically, we have proposed an open-loop stimulation paradigm fashioned as a surrogate CA3 pattern. The evidence brought here indicates that it is possible to recreate this pattern and deploy it in an open-loop stimulation paradigm to control ictogenesis. Compared to periodic pacing, this strategy is straightforward, does not require trial-and-error based fine-tuning, and provides a more efficient and natural way of rectifying state transitions to seizure. 

As electrical stimulation could not be pursued throughout prolonged time scales (i.e., several hours) due to the limited viability of brain slices in time, it remains to be established whether the effects of prolonged stimulation deploying this paradigm may extend beyond the time-frame of stimulus delivery (OFF-stimulation suppression effect). This feature is highly relevant, as it might enable intermittent as opposed to continuous stimulation. In particular, intermittent stimulation could be deployed in a closed-loop approach, based on real-time extraction of signal features that are informative of brain state transitions. Thus, the proposed strategy is expected to improve personalized DBS, both in open-loop and closed-loop approaches. In this regard, in vivo studies in epileptic animals will help validate this paradigm at a higher level of complexity than the in vitro model used here and will provide further insights into long-term effects, as well as the feasibility of implementing epochs of surrogate stimulation in closed-loop DBS. 

As a further insight into a broader perspective, we propose that mimicking relevant temporal dynamics may be conceptually extended to another brain region(s) of interest as well as to other neurological disorders that are currently a candidate for DBS treatment, as a general framework to design physiologically relevant DBS strategies.

## Figures and Tables

**Table 1:** Stimulation parameters for the dataset summarized in Figure 4. *** *p* < 0.0001.

Slice n.	μ	σ	Range [s]	n. Pulses LOGN	n. Pulses 1 Hz	Delta Pulses	Pulse Amplitude [μA]
1	3.90	1.33	1.66–8.65	416	1800	1384 ***	±250
2	1.86	1.36	0.59–5.71	618	1200	582 ***	±300
3	2.76	1.56	1.01–12.27	414	1200	786 ***	±200
4	3.64	1.48	0.57–12.67	290	1200	910 ***	±250
5	1.12	1.27	0.26–2.11	1043	1200	157 ***	±300

**Table 2:** *P_ictal_* in the CTRL phases of the lognormal versus matched periodic stimulation dataset.

Parameter	CTRL1	CTRL2	CTRL3
*P_ictal_*	23.05 ± 3.08	20.89 ± 2.6	21.46 ± 2.44
Ictal amplitude [μV]	858.47 ± 159.8	687.15 ± 110.48	701.7 ± 124.46

**Table 3:** Effect of lognormal and matched periodic stimulation on ictal activity (dataset summarized in Figure 5). *** *p* < 0.0001.

Parameter	Pooled CTRL	STIM LOGN	STIM Matched Periodic
*P_ictal_*	21.8 ± 1.54	2.68 ± 1.05 ***	4.16 ± 1.5 ***
% ictal state reduction	-	87.7 ± 0.4	80.91 ± 6.86

**Table 4:** Stimulation parameters for the dataset summarized in Figure 5.

Slice n.	μ	σ	Range [s]	n. Pulses LOGN	n. Pulses Matched Periodic	Delta PULSES	Pulse Amplitude [μA]
1	1.09	1.22	0.5–2.28	1076	1095	19	±350
2	1.23	1.22	0.61–2.51	960	975	15	±250
3	0.65	1.15	0.39–0.97	1900	1949	49	±200
4	0.59	1.13	0.36–0.9	2012	2027	15	±250
5	1.35	1.31	0.31–3.22	862	888	26	±350
6	0.92	1.28	0.26–2.33	1272	1303	31	±350

## Data Availability

The data presented in this study are openly available in Zenodo at 10.5281/zenodo.6278046, reference number 6278046. The custom MATLAB code used to produce and analyze the experimental data will be provided to any qualified researcher upon reasonable request.
